# An Investigation of Attention to Faces and Eyes: Looking Time Is Task-Dependent in Autism Spectrum Disorder

**DOI:** 10.3389/fpsyg.2018.02629

**Published:** 2018-12-18

**Authors:** Teresa Del Bianco, Noemi Mazzoni, Arianna Bentenuto, Paola Venuti

**Affiliations:** ^1^ODF Lab, Department of Psychology and Cognitive Science, University of Trento, Rovereto, Italy; ^2^Centre for Brain and Cognitive Development, Birkbeck, University of London, London, United Kingdom; ^3^Child Psychopathology Unit, Scientific Institute, IRCCS Eugenio Medea, Bosisio Parini, Italy

**Keywords:** autism spectrum disorder, social gaze, eye-tracking, attention, atypical development

## Abstract

A defective attention to faces and eyes characterizes autism spectrum disorder (ASD), however, the role of contingent information – such as the task instructions – remains still unclear. Our study aimed to investigate the face-orienting response and the subsequent attentive selection in the presence of varying task instructions in individuals with atypical and typical development. Twenty young adults with ASD and 24 young adults with typical development participated in our eye-tracking study. The participants received one of three different instructions at the beginning of each trial and watched scenes of a social interaction. The instructions asked either to find an object (visual-search, VS), to identify which actor was paying attention to the conversation (gaze-reading, GR), or to simply watch the video (free-viewing, FV). We found that the groups did not differ in terms of proportion of first fixations to the face. Nonetheless, average looking time and proportional looking time to faces differed across groups. Furthermore, proportional looking time to faces was task-dependent in the ASD group only, with maximum proportion in the GR and minimum in the VS condition. This result cannot be explained by a lack of an initial bias to orient to the face, since the face-orienting tendency was similar in the ASD and the control group.

## Introduction

The face is the container and the source of rich social information, such as gaze, emotional expressions, and language. Not surprisingly, human adults preferentially orient to and look longer at images of faces compared to other competing stimuli (Palermo and Rhodes, 2007; Shah et al., 2013). This preference is likely regulated by specific properties of the face – primarily, its configuration and contrast polarity, i.e., the reciprocal arrangement of three dark elements, the eyes and the mouth, surrounded by a lighter-colored area (Stein et al., 2011). The same factors influence the orientation to specific features of a face, in particular the eye-gaze (Tipples, 2005). It has been suggested that an *innate* visual preference for faces and eyes subtend this powerful bias (Gliga et al., 2009). In fact, newborns show a strong visual preference for face configuration (Johnson et al., 1991), and infants prefer to look at faces with open eyes (Farroni et al., 2002). This bias has been related to the socio-evolutionary value of the face and the eyes, and it may contribute to the protracted development of the sophisticated human face expertise (Morton and Johnson, 1991; Johnson et al., 2015).

Autism spectrum disorder is a condition of atypical neurodevelopment, characterized by difficulties in social interaction and communication, and restricted interests and behaviors (American Psychiatric Association, 2013). It has been documented that adult people with ASD allocate less attention to faces and their internal features, in particular eyes, and even mouths (Guillon et al., 2014; Chita-Tegmark, 2016). The innate attentive bias that we described above might provoke this attentive deficit (Johnson et al., 2015). This view could explain the specific behavioral and cognitive signatures of ASD, such as the diminished face-processing ability, the disregard for gaze-information, the unawareness and/or the variable degree of difficulty with facial and gaze cues. A major consequence of this deficit could be an insufficient exposure to the face and the eyes during development, not sustained by automatic orienting.

However, longitudinal studies seem to oppose this idea. One study showed an equally powerful attentive bias to faces in infants at-risk (i.e., younger siblings of children with ASD) that later developed the condition and non-at-risk infants during the 1st year of life (Elsabbagh et al., 2013b). In addition, attention to faces appears to decrease only during childhood and adulthood in ASD (Guillon et al., 2016; Kleberg et al., 2017). Specific attention to the eye-gaze is present too in infants at risk of ASD, but starts to decrease earlier, between 2 and 6 months of life, in infants that later develop the condition (Jones and Klin, 2013). These results suggest that the primitive attentive bias for faces and eyes might be intact in ASD early in life, but that attention to the face and the eyes might heterogeneously deteriorate across multiple and possibly divergent developmental paths; therefore, alternative hypotheses have been offered to explain the profound difficulties in face- and gaze-processing that characterize ASD.

An interesting hypothesis focuses on general attentive regulation difficulties that might have cascade effects on the social domain as well as the non-social domain (Elsabbagh et al., 2013b). If face-orienting was subtended by an innate bias, it would only be minimally affected by contextual variation; however, if this function was primarily a specialization of a general attentive ability, it would be expected that factors influencing attentive modulation affect face-orienting as well. In favor of this hypothesis, a set of results highlight that the sensory modality of the contextual information attracting the attention to the face and to the eyes is crucial for the characterization of this deficit in ASD. For instance, it has been reported that the visual preference for the face drops in individuals with ASD when a more refined attentive regulation is required, e.g., when the facial information is conveyed by isolated eye-gaze cues compared to more salient cues, such as global head cues (Thorup et al., 2016), or prioritized by motion cues compared to the static presentation of cues in a sequence (Benson et al., 2009; Fletcher-Watson et al., 2009). Other results suggest that the attention to the face may be even more influenced by contextual enrichment. For instance, individuals with ASD showed diminished face-looking time with realistic video clips and verbal content, e.g., an actor greeting and talking to the participant (Chawarska et al., 2013), and did not increase the fixation duration on the face when it was moving, as opposed to a still portrait, differently from individuals with typical development (Rigby et al., 2016). As a conclusion, differences that might shed light on the nature of the face- and eye-orienting impairment in ASD may involve specific contextual cues that integrate with the attentive regulation function.

Even though gaze and motion cues clearly express a focus of difficulty, to date we lack a complete picture of the alteration of face-orienting and gaze-following as scarce information is available on other types of cues, such as explicit cues. The effect of verbal cues – that often take the form of orders and instructions – on the attention to the face and the eye-gaze might offer a crucial insight about specific patterns of visual exploration in ASD. For instance, in an experiment where participants were explicitly instructed to look for objects, individuals with ASD showed overall shorter fixations compared to individuals with TD (Joseph et al., 2009). This result has been related to a higher mastery of individuals with ASD in the visual processing of objects, compared to individuals with TD. Instead, it may be expected that a task involving the processing of faces and eye-gaze, such as reading facial expressions, might be associated with longer fixations, due to more difficult processing (Baron-Cohen et al., 1995); however, this effect has not been investigated yet with the eye-tracking technique.

### Aim of the Current Study

This study aimed to gain additional insight into the attentional processes involved in the lack of specific attention to the faces and the eye-gaze in ASD. We believe that it is crucial to clarify whether an innate attentive bias for faces is impaired, or the lack of preference for faces is affected by a general-attentive dysregulation. In fact, the contribution of one of these two factors may give a very different outcome. In order to achieve this goal, we measured the face-orienting tendency and the face-looking time in young adults with and without ASD using realistic, dynamical stimuli representing a simple social interaction. Additionally, we included explicit instructions that required that the participant completed a visual-search and a mentalization task, conditions that have not been directly explored with the eye-tracking technique. In our view, a lack of specific innate bias would affect the attention to the face, while deviances regarding other areas of interest would be non-significant. We expected face-orienting to differ between the groups. Regarding the effect of the explicit instructions, we expected that the task instructions might have opposite effects in participants with TD and ASD. Participants with ASD might be less attracted to the face and display shorter fixations’ duration compared to participants with TD, even when the instruction focuses on a task that prioritizes the information coming from the face and the eye-gaze. On the other hand, if the alteration lies on a general impairment of attention, we may observe a similar pattern in both conditions simply because the instruction requires a certain degree of attentive regulation. This effect may also be generalized to AOIs other than the face and would regard the visual-search condition too.

## Materials and Methods

### Participants

Twenty-four young adults with typical development (TD) and 20 young adults with a diagnosis of “High Functioning Autism” (13) or “Asperger Syndrome” (7) participated the study. Experienced clinicians established that the participants met the criteria for ASD as specified in the Diagnostic and Statistical Manual IV (DSM-IV; American Psychiatric Association, 1994) or DSM-5 (American Psychiatric Association, 2013), or the Autism Diagnostic Observation Schedule (Lord et al., 2000), or the Autism Diagnostic Interview (Lord et al., 1994). The essential information about the participants is reported in Table 1. Wilcoxon tests indicate that participants did not differ in terms of age (*W* = 228, *p*-value = 0.58), the Intelligence Quotient (IQ) as measured with the Raven Matrices (*W* = 90.5, *p*-value = 0.07) and the Socio-Economical Score (SES; *W* = 179, *p*-value = 0.62). Additionally, the IQ of participants with ASD was assessed with Wechsler Scales: as the verbal sub-quotient of the participants with ASD lied within the normative range, we expected an optimal reception of the verbal instructions and included all of them in the analysis. Participants with TD were recruited at the University of Trento; participants with ASD were recruited at the “Laboratory of Diagnosis, Observation, and Education” (ODFLab) of the University of Trento. Written informed consent was obtained from all the participants in accordance with the Declaration of Helsinki.

**Table 1:** Means and standard deviations of Age, SES and IQ (ND, neurodevelopment; SES, socio-economical score; IQ, intelligence quotient; ♀ = female; N = number; M = mean; SD = standard deviation).

ND	Age [*M* (*SD*)]	SES^1^ [*M* (*SD*)]	♀(*N*)	IQ Raven [*M* (*SD*)]	Total IQ Wechsler [*M* (*SD*)]	Verbal IQ Wechsler [*M* (*SD*)]	Performance IQ Wechsler [*M* (*SD*)]
TD	22.4 (3)	42.1 (11.4)	8	122.4 (8.1)	NA	NA	NA
ASD	22.1 (3.8)	43.8 (14.4)	0	118 (10)	96.11 (11.6)	100 (15.6)	101 (14.4)


*^1^Socio-economical score, calculated using the Four-Factor Index of Social Status (Hollingshead, 1975). The sample represented a medium status in the Italian population (Venuti and Senese, 2007)*.

### Tools

#### Apparatus

We used a Tobii T120 eye-tracker (Tobii Technology, Stockholm), with a sampling rate of 60 Hz. The integrated monitor had a resolution of 1280^∗^1024 and a size of 17″. The experiment was designed and run through the software Ogama (Vosskühler et al., 2008). For collecting the participant’s answers, we used a Python script.

#### Stimuli

The stimuli consisted of 24 10-s videos, displaying 3 actors seated in front of a neutral wall. The central actor was always a female, while in half of the videos the actors on the sides were both males and the other half they were both females. The central model had only her back visible, and she pronounced a predefined sentence in Italian (i.e., “I will go home next Tuesday. I am going to University with the whole family.”). When the central actor started to talk, the two models on the sides shifted their gazes either toward/away from the central model. The other two actors were facing-forward and one of them wore a pen on his/her shirt. The position of the facing-forward actors, the direction of the eye-gaze toward and away from the central actor, and the position of the pen were counterbalanced across the experiment.

During each block, a 7-s instruction preceded the onset of the video and a 7-s answer screen followed the video. The total duration of one block (including instruction, video and answer screen) was 24 s. The three types of instruction were:

(1) Simply watching the video (“Now, simply watch the video”; free-watching condition, FV).
(2) Finding the specified object located on the body of one of the models (“Now answer the question: Who has the pen?”; visual search condition, VS).
(3) Identifying who is listening by using eye-gaze direction information (“Now answer the question: Who is listening?”; gaze-reading condition, GR).

The correct answers consisted in indicating the side of the model wearing the pen on his/her body (VS condition) or that shifted his/her eye-gaze toward the central model (GR condition) by pressing the key A (left) and L (right). The A and the L keys were covered with a white tag.

**FIGURE 1:**
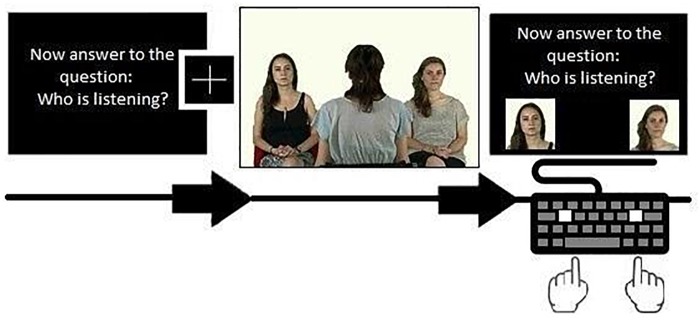
Example of the sequence composed by instruction, fixation cross, video and answer screen. Written informed consent was obtained from the 3 models to authorize the publication of this photograph as a representation of the stimulus used in the experiment.

Each question was repeated on the answer screen above two photographs of the actor, located at the sides of the screen. Each instruction was repeated 8 times in a randomized order, for a total of 24 blocks per participants. Each trial started with one of these instructions, for a total of 8 FV, 8 VS, and 8 GR conditions. For a graphical representation of the stimuli presentation, see Figure 1.

### Procedure

The participant sat in from of the eye-tracker and the keyboard in a homogeneously well-lit room. The experimenter explained the calibration procedure and instructed the participants to follow the instructions before each video and to press one of the two specified keys to choose an answer when displaying the answer screen. The keys were selected to be widely apart (L and A, respectively, at the extreme right and left of the Italian keyboard). The keys were marked by a white label, highly contrasted with the black keyboard (see Figure 1). The participant was instructed to press the key corresponding to the position of the actor on the answer screen (right or left). After instructing the participant, the experimenter sat behind a curtain and monitored the participant’s gaze.

Before starting the experiment, the participants performed two practicing blocks without recording eye movements. Subsequently, the experimenter started the 5-points calibration procedure, consisting in a red ball moving between the edges and the center of the screen. The calibration was accepted when all the positions had been sampled (on average, no more than 2 attempts were needed for each participant). The proportion of first fixations landing on the face, the average fixation duration on the face and on the body, the proportional looking time on face, and the percentage of correct responses were calculated.

### Data Analysis

#### Preliminary Analysis

We pre-processed the data using the standard fixation filter of Ogama (distance threshold = 35 pixels, samples minimum value = 10). Total fixation durations were calculated within 6 predefined AOIs, (face and body of the models, central model and background), drawn on the stimuli and aggregated in two groups (faces and bodies). The following preliminary and main analysis were carried out in R (R Core Team, 2015).

The percentage of correct responses given with the keyboard over the total number of responses was calculated for each subject (accuracy hereafter). By comparing the accuracy between groups and conditions, we ensured that participants from both groups understood the task correctly and were able to deliver a correct response. In fact, Wilcoxon Tests did not show any significant difference in accuracy between participants with ASD and TD (general accuracy: ASD = 87.9%, standard deviation = 12.8, TD = 88.8%, standard deviation = 12.4, *W* = 224, *p*-value = 0.7. VS: ASD = 81.2%, standard deviation = 24.1, TD = 81.2%, standard deviation = 24.1, *W* = 230.5, *p*-value = 0.82. GR: ASD = 88.8%, standard deviation = 16.1, TD = 85.9%, standard deviation = 20.6, *W* = 225.5, *p*-value = 0.71). Furthermore, we analyzed a general measure that is negatively correlated with search efficiency, the number of fixations within the AOIs (Holmqvist et al., 2011). The number of fixations on the face across conditions did not differ between groups (FV: *W* = 178.5, *p*-value = 0.30; GR: *W* = 167, *p*-value = 0.18; VS: *W* = 150.5, *p*-value = 0.08) or within groups (ASD: GR vs. VS, *W* = 112.5, *p*-value = 0.01 – not resisting to Bonferroni correction -, FV vs. VS, *W* = 255, *p*-value = 0.49, GR vs. FV, *W* = 147, *p*-value = 0.15), suggesting that the level of processing difficulty of the gaze-information was similar across groups and tasks. However, the groups differed significantly for the number of fixations on body in the VS condition (*W* = 331.5, *p*-value = 0.005), that was higher than in the other conditions in the ASD group (VS vs. FV, *W* = 111.5, *p*-value = 0.017, VS vs. GR, *W* = 352, *p*-value = < 0.001).

The average data loss (as measured by the output “Percentage of Samples Out of the Screen” of the Ogama software) within the duration of the movies was very low, with an average of 0.01 (standard deviation = 0.1) in the TD group and 1.6 (standard deviation = 6.5) in the ASD group. The percentage of data loss was compared through the Wilcoxon Test and did not differ between groups (*W* = 271, *p*-value = 0.36). The data loss had inter-subject minimal variation, with a minimum *z*-score of -0.17 and a maximum *z*-score of 0.08 across the two groups.

#### Main ***A***nalysis

As the variables were not normally distributed, we performed all the analysis with non-parametric tests (Wilcoxon Signed Rank Tests). To test whether the faces would primarily attract the attention of both groups, we calculated the proportion of first fixations landing on the face (FF%). To calculate the FF%, we selected only those trials where the eye position was recorded on the AOI “Center” before the onset of the first gaze shift (see Table 2; for mean proportions of FF%, see Table 3). Participants who displayed less than 3 valid trials were excluded from the subsequent analysis, thus resulting in a final sample of 39 participants (16 from the ASD group, 23 from the TD group). We divided the total numbers of valid trials where the first fixation landed on the AOI “Face” by the total numbers of valid trials where the first fixation landed on any of the other AOIs, i.e., body, central model and background (FF% means and standard deviations are reported in Tables 4, 5). We then compared the FF% to the probability of hitting the AOI “Face” by chance (1/total N of independent AOIs = 0.2) and performed group comparisons. A proportion significantly higher than chance indicate that a bias to shift the eye-movement from the fixation center to the face exist. An equal proportion in both groups may indicate that participants with and without ASD showed a similar bias to direct their first fixation to the face. Additionally, we compared the number of trials where the first fixation landed on the face compared to the number of trials where the first fixation landed on any other AOI (with a generalized linear mixed model).

**Table 2:** Number of valid trials per condition for each participants’ group (ND, neurodevelopment; ASD, autism spectrum disorder; TD, typical development; FV, free-viewing; VS, visual search; GR, gaze-reading; N, number; M, mean; SD, standard deviation).

ND	Condition	*N* of Valid Trials [*M* (*SD*)]
ASD	FV	6.8 (1.42)
	VS	6.47 (1.64)
	GR	6.44 (1.79)
TD	FV	6.3 (1.74)
	VS	6.87 (1.58)
	GR	6.35 (1.61)


**Table 3:** Mean proportion and standard deviations of FF% by group and condition (ND, neurodevelopment; ASD, autism spectrum disorder; TD, typical development; FV, free-viewing; VS, visual search; GR, gaze-reading; N, number; M, mean; SD, standard deviation).

ND	Condition	*M* (*SD*)
ASD	FV	0.44 (0.21)
	VS	0.42 (0.27)
	GR	0.52 (0.27)
TD	FV	0.37 (0.27)
	VS	0.34 (0.26)
	GR	0.57 (0.23)


**Table 4:** output of the mixed model analyzing the number of trials where the first fixation landed on the face in the two groups.

Fixed effect	Estimate (logit)	Standard error	*Z*-Value	*P*-value
ND: ASD	1.41	0.35	3.98	<0.001(*)
ND: TD	1.49	0.31	4.67	<0.001(*)
Condition: VS	–1.33	0.28	–4.65	<0.001(*)
Condition: GR	0.49	0.31	1.58	0.11
**Interactions**
ND^∗^Condition: VS	–0.14	0.37	–0.38	0.70
ND^∗^Condition: GR	0.55	0.44	1.25	0.20


*Estimates that are significantly different from 0 are marked with a (^∗^). The estimates are expressed in the logit scale and can be converted to proportions with the formula exp(logit)/[1 + exp(logit)]*.

**Table 5:** Mean FD and standard deviations in seconds on face and body by group and condition (ND, neurodevelopment; ASD, autism spectrum disorder; TD, typical development; FV, free-viewing; VS, visual search; GR, gaze-reading; N, number; M, mean; SD, standard deviation).

ND	AOI	Condition	*M* (*SD*)
ASD	Face	FV	4.06 (2.16)
		VS	3.77 (1.95)
		GR	5.54 (1.98)
	Body	FV	1.27 (0.96)
		VS	2.70 (1.50)
		GR	0.79 (0.65)
TD	Face	FV	2.87 (1.20)
		VS	2.67 (1.40)
		GR	3.78 (1.40)
	Body	FV	1.29 (0.74)
		VS	1.47 (0.78)
		GR	0.63 (0.48)


For exploring the effect of task instructions, we examined two aggregated measures: the average fixation duration on the face and on the body (FD), and the proportional looking time on face (LT%, calculated as the Total Fixation Duration on the Face divided by the Total Fixation Duration on all the AOIs; means and standard deviations of LT% are reported in Table 6). We compared the FD on the body and the face, and the LT% on face between and within groups through Wilcoxon Tests. Both variables account for the adaptation of eye-movements to instructions (Holmqvist et al., 2011); furthermore, the proportional looking time accounts also for idiosyncratic scanning differences (Fu et al., 2012).

**Table 6:** Mean LT% on face and standard deviations by group and condition (ND, neurodevelopment; ASD, autism Spectrum disorder; TD, typical development; FV, free-viewing; VS, visual search; GR, gaze-reading; N, number; M, mean; SD, standard deviation).

ND	Condition	*M* (*SD*)
ASD	FV	0.72 (0.2)
	VS	0.59 (0.16)
	GR	0.87 (0.12)
TD	FV	0.91 (0.15)
	VS	0.91 (0.14)
	GR	0.94 (0.10)


All the reported comparisons have been selected through Bonferroni Correction (*p*-value < 0.05/*N* of comparisons).

## Results

### Proportion of First Looks From Center to the Face (FF%)

**FIGURE 2:**
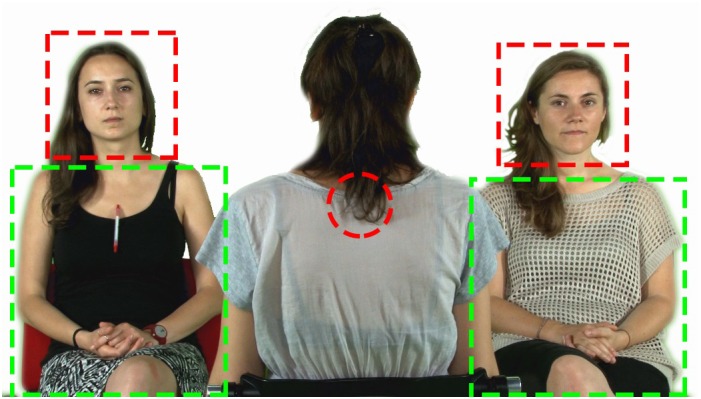
Screenshot of one of the video frames with the superimposed AOIs (face, body, centre of the screen).

Multiple Wilcoxon tests revealed that FF% was above chance in all groups and conditions (FV: ASD: *W* = 115, *p*-value = 0.001, TD: *W* = 216, *p*-value = 0.009; VS: ASD: *W* = 108, *p*-value = 0.003; GR: ASD: *W* = 134, *p*-value < 0.001, TD: *W* = 272, *p*-value < 0.001). The result of TD participants in Condition 2 was significant but did not resist to Bonferroni Correction (*p*-value > 0.01). The groups did not differ in terms of FF% across conditions (FV: *W* = 199.5, *p*-value = 0.42; VS: *W* = 209.5, *p*-value = 0.27; GR: *W* = 156, *p*-value = 0.43). The comparison between conditions within each group carried out with Kruskal–Wallis Test was not significant for the ASD group (Chi-squared = 1.2, *p*-value = 0.52); the same test turned out significant for the TD group (Chi-squared = 9.5, *p*-value = 0.008). Additional Wilcoxon tests revealed that the VS conditions significantly differed from both FV and GR conditions in TD with a lesser proportion of first fixations to the face (VS vs. GR: *W* = 33, *p*-value = 0.001; VS vs. FV: *W* = 5, *p*-value = 0.0001). The results concerning FF% are displayed in Figure 2.

### Number of Trials Where the First Fixation Landed on the Face

**FIGURE 3:**
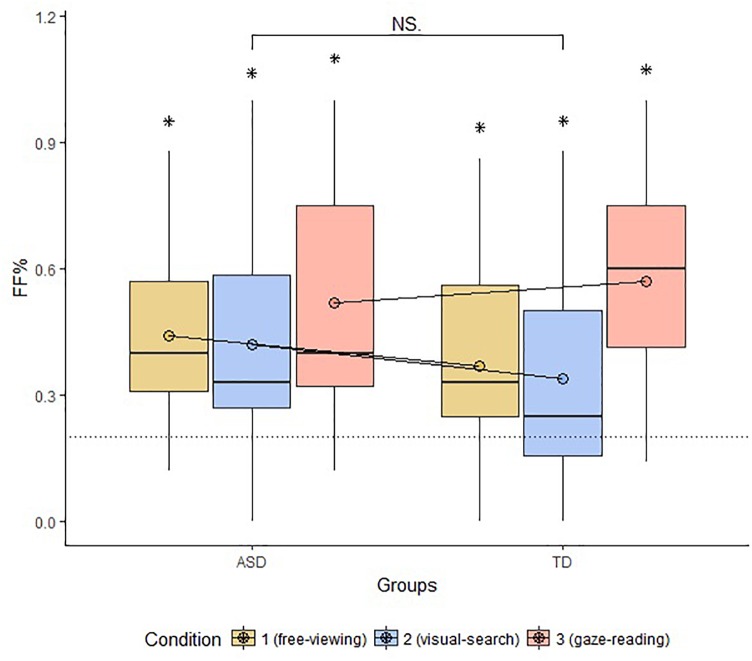
Boxplot of FF% across groups and conditions. The circles drawn on the box represent the mean FF% by group and condition. The dotted line marks the chance level of hitting the AOI face first. The bracket indicates general comparisons between and within groups (FF%, proportion of first fixations to face; ASD, autism spectrum disorder; TD, typical development; ^∗^, parameter significantly above chance level; NS, non-significant comparison).

Our generalized linear model included group, condition and their interaction as predictors of the number of trials where the first fixation landed on the face. The model allowed for random intercepts for each subject, and no fixed intercept. The model showed that participants pertaining to both groups significantly fixated the face first, compared to other AOIs, in the majority of trials – around 80% (ASD: estimate = 1.41, standard error = 0.35, *z*-value = 3.98, *p*-value < 0.001; TD: estimate = 1.49, standard error = 0.31, *z*-value = 4.67, *p*-value < 0.001). The model also indicates that the probability of hitting the face was significantly lower in the VS condition. However, the interaction between the condition and the group were not significant (see Tables 4, 5 and Figure 3 for further details), confirming that the groups did not differ across conditions.

### Average Fixation Duration (FD)

**FIGURE 4:**
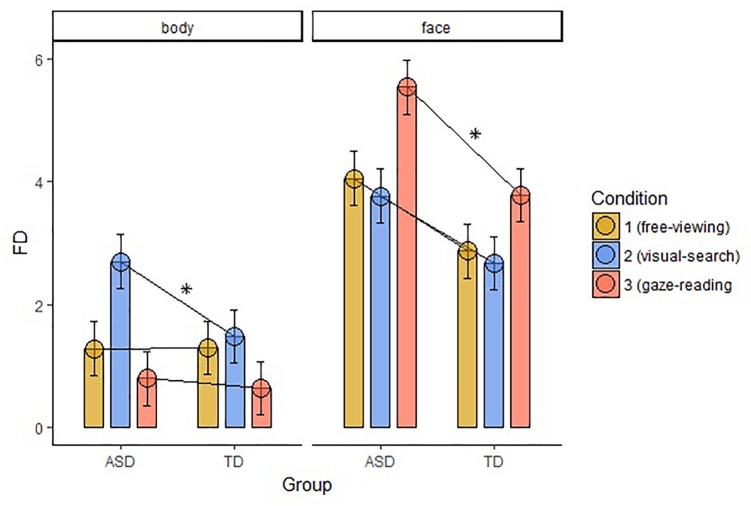
Average FD within the two groups of AOIs, body and face, across groups and conditions (FD, fixation duration; ASD, autism spectrum disorder; TD, typical development; ^∗^, significant comparison).

Fixation duration (FD) on Body differed significantly between the groups in the VS condition (*W* = 369, *p*-value = 0.001), with longer FD in the ASD group. FD on Face was significantly different between the groups in the GR condition (*W* = 388, *p*-value < 0.001), with longer FD in the ASD group. We found no significant correlations between the FD and the IQ level of the participants in both groups. The results are displayed in Figure 4.

### Proportional Looking Time on Face Compared to the Other AOIs (LT%)

**FIGURE 5:**
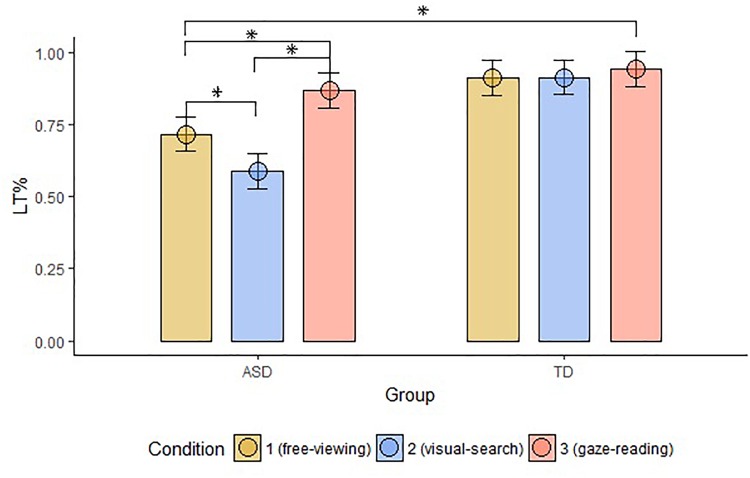
Mean LT% on Face, compared to the other AOIs, across groups and conditions. The top bracket indicates between group comparisons. The additional three brackets indicate within groups comparisons (LT%, proportional looking-time on face; ASD, autism spectrum disorder; TD, typical development; ^∗^, significant comparison).

The groups differed in terms of LT% across conditions (FV: *W* = 87, *p*-value < 0.001; VS: *W* = 36, *p*-value < 0.001; GR: *W* = 113, *p*-value = 0.002). Within-group comparisons showed that LT% significantly differed across conditions in the ASD group only (significant alpha-value < 0.01; ASD, FV vs. VS: *W* = 157, *p*-value = 0.002, FV vs. GR: ASD: *W* = 13, *p*-value < 0.001, VS vs. GR: ASD: *W* = 13, *p*-value < 0.001; TD, FV vs. VS: W = 34, *p*-value = 0.96, FV vs. GR: W = 9, *p*-value = 0.03, VS vs. GR: *W* = 8, *p*-value = 0.09), as shown in Figure 5.

## Discussion

In the present study, we investigated the state of face-orienting and face-looking time in young adults with ASD using realistic stimuli and explicit task instructions. In sum, our results show that:

(1) Face-orienting was above chance in participants with ASD, irrespective of the type of instruction; the groups did not differ in terms of FF% or in terms of the number of trials where the first fixation landed on the face.
(2) Participants with ASD displayed longer fixation times on task-relevant areas of interest – face and body. Furthermore, they displayed a greater number of fixations on the body only
(3) The proportional looking time on the face was task-dependent in the ASD group, with maximum proportion in the GR condition, and minimum proportion in the VS condition. The same measure did not vary between conditions in the TD group.

The first set of results is in contrast with the renowned evidence of people with ASD looking less at faces (Klin et al., 2002; Chawarska et al., 2010); this discrepancy might be explained by the fact that our stimuli represented a fairly simple situation (compared for instance to the scenes of the movie “Who is afraid of Virginia Woolf?” used by Klin et al., 2002). Furthermore, all the participants in the ASD group were high functioning and may have developed strategies or compensatory mechanism to obviate face-processing difficulty. Nonetheless, our result is in line with the evidence that face-orienting abilities are not always impaired in individuals with high-functioning ASD (Shah et al., 2013; Elsabbagh et al., 2014). Considering that the face-orienting bias is (1) documented in infants at risk of ASD (Elsabbagh et al., 2013b), (2) heterogeneously impaired in children with ASD (Chawarska et al., 2013), and (3) correlates with face-processing abilities (de Klerk et al., 2014), we may conclude from our result that face-orienting may either deteriorate in certain subgroups of children with ASD or endure a developmental delay, but it possibly recovers and/or establish compensatory mechanisms in adulthood (Belmonte and Yurgelun-Todd, 2003; New et al., 2010; Sheth et al., 2010). A putative mechanism might be the progressive specialization of the face-sensitive areas, whose specialization is not fully accounted by an innate preparedness (Johnson, 2011). Indeed, according to the Interactive Specialization view (Johnson, 2011), accumulating experience with faces is crucial for the refinement of automatic face-orienting mechanisms. In ASD, while the automatic ability to orient to faces is intact, several other symptoms can impede the massive input of face experience and prevent the development of the deputed neural areas and, hence, the development of refined face-processing skills (Johnson et al., 2005). However, additional cognitive resources, an improved management of symptoms in high functioning individuals, and/or ongoing intensive therapy, can counterbalance the initial lack of experience and start a progressive recovery of face-processing skills.

The second set of results is in line with the hypothesis that difficulties in face encoding and in gaze-processing correlates with a prolonged fixation duration on the face in individuals with ASD, as it has been previously reported (Elsabbagh et al., 2013a). However, we did not observe any difference in the accuracy of the responses. Furthermore, the longer fixation duration was not limited to the faces in the GR condition, but involved the body in the VS condition too, thus excluding a face-processing difficulty. Notably, the significant differences were limited to those AOIs that were relevant to the task (Body in VS condition, Face in GR condition).

**FIGURE 6:**
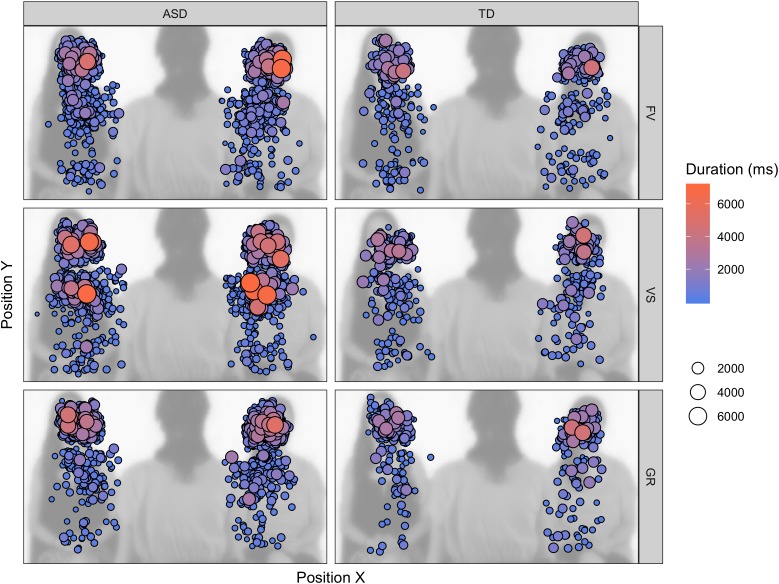
Fixation map on the task-relevant AOIs (faces, bodies) across the experiment, superimposed on one of the video frames. The size and color of the fixation indicate the duration of the individual fixation.

The longer FD on task-relevant areas and the variable amount of face LT% depending on the instruction could be explained with participants with ASD taking more time to correctly elaborate the stimulus and to extract from the stimuli the task-relevant information or sticking more to the task and being less distracted by the other available AOIs. In the first case, participants with ASD would perform a higher number of longer fixations because they need more time to elaborate the information contained in the stimulus to reach the same level of accuracy of participants with TD. In the second case, participants with ASD would perform longer but an equal number of fixations because they have difficulty to disengage from the AOI brought into focus by the instruction. With regard to the face, we cannot overrule one of these two explanations, as we did not find increased number of fixations; however, the AOI drawn on the face is relatively small, and all the information conveyed by its elements can be explored without increasing the number of eye-movements. On the contrary, the result regarding the body suggests that individuals with ASD sampled a higher number of positions for longer periods of time for succeeding the visual search (see fixation map in Figure 6). It is also noticeable that participants with ASD shifted their first fixation on the face anyway in the VS condition, while participants with TD did not prioritize the face in this condition (i.e., FF% is not significantly above chance) – suggesting that participants with ASD might have had less task efficiency. One of the implications of this results may be that, when their attention is explicitly drawn to objects, persons with ASD may take longer for elaborating the stimuli and end up disregarding other visual items – faces and their components included, such as the eyes. This fact may be problematic for people with ASD, as extrinsic events often disturb social interactions and social partners highlight external objects with gestures and utterances. Once an object captivates their attention, a particularly difficult task may arise for people with ASD, as they need time to elaborate and flounder to shift their attention back to the face and the eyes, with all that this implies. Moreover, this peculiar attentive style may be reconnected to the description of the defective “zooming-out” of the attentional focus and the overly circumscribed orienting tendencies in individuals with ASD (Robertson et al., 2013; Ronconi et al., 2013).

## Conclusion

In the current study, we did not record any difference in face-orienting in the presence of explicit instructions and with dynamical, realistic video clips of a social interaction: this result suggests that the orientation to faces and eyes might undergo compensatory mechanisms that are acquired and refined thanks to the accumulation of experience with human social partners. On the other hand, the average looking time was prolonged on task-relevant areas not limited to the face and the eyes in all the experimental conditions – observation that could be explained by general processing difficulties in young adults with ASD. The major implication of this interpretation is that this alteration of a domain-general difficulty might have even more massive cascade effects compared to the alteration of a domain-specific function, such as gaze-processing. In fact, a child that that is not able to “zoom-out” might miss interaction opportunity, overly focus on complex environmental stimuli, and even become upset if the prolonged focus of attention involves distressing stimuli. This picture overlaps with existing reports of the orienting tendencies of individuals with ASD, i.e., less responsive and prone to distress (Zwaigenbaum et al., 2005). Future research could investigate the transversal effects of these defects. We believe that our result (of task-dependent fixation with two tasks that involved completely different goals) might constitute a first example of this case.

## Ethics Statement

This study was carried out in accordance with the recommendations of the Ethics Committee of the University of Trento with written informed consent from all subjects. All subjects gave written informed consent in accordance with the Declaration of Helsinki. The protocol was approved by the Ethics Committee of the University of Trento.

## Author Contributions

TDB designed the experiments, recruited the participants and collected the data, carried out the statistical analysis and wrote the first draft of the manuscript, under the supervision of AB and PV. NM and AB helped recruiting the participants and collecting the data. TDB, NM, AB, and PV actively discussed the results and outlined the interpretation together. TDB finalized the draft that was later reviewed by NM, AB, and PV.

## Conflict of Interest Statement

The authors declare that the research was conducted in the absence of any commercial or financial relationships that could be construed as a potential conflict of interest.

## References

[B1] American Psychiatric Association (1994). *Diagnostic and Statistical Manual of Mental Disorders, (DSM IV)*, 4th Edn, Vol. 915 Washington, DC: American Psychiatric Association

[B2] American Psychiatric Association (2013). *The Diagnostic and Statistical Manual of Mental Disorders (V).* Arlington, VA: APA 10.1176/appi.books.9780890425596

[B3] Baron-Cohen S. Campbell R. Karmiloff-Smith A. Grant J. Walker J. (1995). Are children with autism blind to the mentalistic significance of the eyes? *Br. J. Dev. Psychol.* 13 379–398. 10.1111/j.2044-835X.1995.tb00687.x 9084124

[B4] Belmonte M. K. Yurgelun-Todd D. A. (2003). Functional anatomy of impaired selective attention and compensatory processing in autism. *Cogn. Brain Res.* 17 651–664. 10.1016/S0926-6410(03)00189-7 14561452

[B5] Benson V. Piper J. Fletcher-Watson S. (2009). Atypical saccadic scanning in autistic spectrum disorder. *Neuropsychologia* 47 1178–1182. 10.1016/j.neuropsychologia.2008.11.019 19094999

[B6] Chawarska K. Macari S. Shic F. (2013). Decreased spontaneous attention to social scenes in 6-month-old infants later diagnosed with ASD. *Biol. Psychiatry* 74 195–203. 10.1016/j.biopsych.2012.11.022 23313640 PMC3646074

[B7] Chawarska K. Volkmar F. Klin A. (2010). Limited attentional bias for faces in toddlers with autism spectrum disorders. *Arch. Gen. Psychiatry* 67 178–185. 10.1001/archgenpsychiatry.2009.194 20124117 PMC4871149

[B8] Chita-Tegmark M. (2016). Social attention in ASD?: a review and meta-analysis of eye-tracking studies. *Res. Dev. Disabil.* 48 79–93. 10.1016/j.ridd.2015.10.011 26547134

[B9] de Klerk C. C. J. M. Gliga T. Charman T. Johnson M. H. (2014). Face engagement during infancy predicts later face recognition ability in younger siblings of children with autism. *Dev. Sci.* 17 596–611. 10.1111/desc.12141 24314028

[B10] Elsabbagh M. Bedford R. Senju A. Charman T. Pickles A. Johnson M. H. (2014). What you see is what you get: contextual modulation of face scanning in typical and atypical development. *Soc. Cogn. Affect. Neurosci.* 9 538–543. 10.1093/scan/nst012 23386743 PMC3989131

[B11] Elsabbagh M. Fernandes J. Jane Webb S. Dawson G. Charman T. Johnson M. H. (2013a). Disengagement of visual attention in infancy is associated with emerging autism in toddlerhood. *Biol. Psychiatry* 74 189–194. 10.1016/j.biopsych.2012.11.030 23374640 PMC3715700

[B12] Elsabbagh M. Gliga T. Pickles A. Hudry K. Charman T. Johnson M. H. (2013b). The development of face orienting mechanisms in infants at-risk for autism. *Behav. Brain Res.* 251 147–154. 10.1016/j.bbr.2012.07.030 22846849 PMC3730054

[B13] Farroni T. Csibra G. Simion F. Johnson M. H. (2002). Eye contact detection in humans from birth. *Proc. Natl. Acad. Sci. U.S.A.* 99 9602–9605. 10.1073/pnas.152159999 12082186 PMC123187

[B14] Fletcher-Watson S. Leekam S. R. Benson V. Frank M. C. Findlay J. M. (2009). Eye-movements reveal attention to social information in autism spectrum disorder. *Neuropsychologia* 47 248–257. 10.1016/j.neuropsychologia.2008.07.016 18706434

[B15] Fu G. Hu C. S. Wang Q. Quinn P. C. Lee K. (2012). Adults scan own- and other-race faces differently. *PLoS One* 7:37688. 10.1371/journal.pone.0037688 22675486 PMC3365898

[B16] Gliga T. Elsabbagh M. Andravizou A. Johnson M. H. (2009). Faces attract infants’ attention in complex displays. *Infancy* 14 550–562. 10.1080/15250000903144199 32693531

[B17] Guillon Q. Hadjikhani N. Baduel S. Rogé B. (2014). Visual social attention in autism spectrum disorder: insights from eye tracking studies. *Neurosci. Biobehav. Rev.* 42 279–297. 10.1016/j.neubiorev.2014.03.013 24694721

[B18] Guillon Q. Rogé B. Afzali M. H. Baduel S. Kruck J. Hadjikhani N. (2016). Intact perception but abnormal orientation towards face-like objects in young children with ASD. *Sci. Rep.* 6:22119. 10.1038/srep22119 26912096 PMC4766445

[B19] Hollingshead A. B. (1975). *Four Factor Index of Social Status.* New Haven, CT: Privately Printed.

[B20] Holmqvist K. Nyström M. Andersson R. Dewhurst R. Jarodzka H. van de Weijer J. (2011). *Eye Tracking: A Comprehensive Guide to Methods and Measures.* Oxford: Oxford University Press.

[B21] Johnson M. H. (2011). Interactive specialization: a domain-general framework for human functional brain development? *Dev. Cogn. Neurosci.* 1 7–21. 10.1016/j.dcn.2010.07.003 22436416 PMC6987575

[B22] Johnson M. H. Dziurawiec S. Ellis H. Morton J. (1991). Newborns’ preferential tracking of face-like stimuli and its subsequent decline. *Cognition* 40 1–19. 10.1016/0010-0277(91)90045-6 1786670

[B23] Johnson M. H. Griffin R. Csibra G. Halit H. Farroni T. de Haan M. (2005). The emergence of the social brain network: evidence from typical and atypical development. *Dev. Psychopathol.* 17 599–619. 10.1017/S0954579405050297 16262984 PMC1464100

[B24] Johnson M. H. Senju A. Tomalski P. (2015). The two-process theory of face processing: modifications based on two decades of data from infants and adults. *Neurosci. Biobehav. Rev.* 50 169–179. 10.1016/j.neubiorev.2014.10.009 25454353

[B25] Jones W. Klin A. (2013). Attention to eyes is present but in decline in 2-6-month-old infants later diagnosed with autism. *Nature* 504 427–431. 10.1038/nature12715 24196715 PMC4035120

[B26] Joseph R. M. Keehn B. Connolly C. Wolfe J. M. Horowitz T. S. (2009). Why is visual search superior in autism spectrum disorder? *Dev. Sci.* 12 1083–1096. 10.1111/j.1467-7687.2009.00855.x 19840062 PMC12049234

[B27] Kleberg J. L. Thorup E. Falck-Ytter T. (2017). Visual orienting in children with autism: hyper-responsiveness to human eyes presented after a brief alerting audio-signal, but hyporesponsiveness to eyes presented without sound. *Autism Res.* 10 246–250. 10.1002/aur.1668 27454075 PMC5324587

[B28] Klin A. Jones W. Schultz R. Volkmar F. Cohen D. (2002). Visual fixation patterns during viewing of naturalistic social situations as predictors of social competence in individuals with autism. *Arch. Gen. Psychiatry* 59 809–816. 10.1001/archpsyc.59.9.809 12215080

[B29] Lord C. Risi S. Lambrecht L. Cook E. H. Jr. Leventhal B. L. DiLavore P. C. (2000). The autism diagnostic observation schedule-generic: a standard measure of social and communication deficits associated with the spectrum of autism. *J. Autism Dev. Disord.* 30 205–223. 10.1023/A:1005592401947 11055457

[B30] Lord C. Rutter M. Le Couteur A. (1994). Autism diagnostic interview-revised: a revised version of a diagnostic interview for caregivers of individuals with possible pervasive developmental disorders. *J. Autism Develop. Disord.* 24 659–685. 10.1007/BF02172145 7814313

[B31] Morton J. Johnson M. H. (1991). CONSPEC and CONLERN: a two-process theory of infant face recognition. *Psychol. Rev.* 98 164–181. 10.1037/0033-295X.98.2.164 2047512

[B32] New J. J. Schultz R. T. Wolf J. Niehaus J. L. Klin A. German T. C. (2010). The scope of social attention deficits in autism: prioritized orienting to people and animals in static natural scenes. *Neuropsychologia* 48 51–59. 10.1016/J.NEUROPSYCHOLOGIA.2009.08.008 19686766 PMC6102729

[B33] Palermo R. Rhodes G. (2007). Are you always on my mind? A review of how face perception and attention interact. *Neuropsychologia* 45 75–92. 10.1016/j.neuropsychologia.2006.04.025 16797607

[B34] R Core Team (2015). *R: A Language and Environment For Statistical Computing.* Vienna: R Foundation for Statistical Computing.

[B35] Rigby S. N. Stoesz B. M. Jakobson L. S. (2016). Gaze patterns during scene processing in typical adults and adults with autism spectrum disorders. *Res. Autism Spectr. Disord.* 25 24–36. 10.1016/j.rasd.2016.01.012

[B36] Robertson C. E. Kravitz D. J. Freyberg J. Baron-Cohen S. Baker C. I. (2013). Tunnel vision: sharper gradient of spatial attention in autism. *J. Neurosci.* 33 6776–6781. 10.1523/JNEUROSCI.5120-12.2013 23595736 PMC3640213

[B37] Ronconi L. Gori S. Ruffino M. Molteni M. Facoetti A. (2013). Zoom-out attentional impairment in children with autism spectrum disorder. *Cortex* 49 1025–1033. 10.1016/j.cortex.2012.03.005 22503282

[B38] Shah P. Gaule A. Bird G. Cook R. (2013). Robust orienting to protofacial stimuli in autism. *Curr. Biol.* 23 R1087–R1088. 10.1016/j.cub.2013.10.034 24355781 PMC3898081

[B39] Sheth B. R. Liu J. Olagbaju O. Varghese L. Mansour R. Reddoch S. (2010). Detecting social and non-social changes in natural scenes: performance of children with and without autism spectrum disorders and typical adults. *J. Autism. Dev. Disord.* 41 434–446. 10.1007/s10803-010-1062-3 20614172

[B40] Stein T. Peelen M. V. Sterzer P. (2011). Adults’ awareness of faces follows newborns’ looking preferences. *PLoS One* 6:29361. 10.1371/journal.pone.0029361 22216259 PMC3244447

[B41] Thorup E. Nyström P. Gredebäck G. Bölte S. Falck-Ytter T. (2016). Altered gaze following during live interaction in infants at risk for autism: an eye tracking study. *Mol. Autism* 7:12. 10.1186/s13229-016-0069-9 26819699 PMC4729153

[B42] Tipples J. (2005). Orienting to eye gaze and face processing. *J. Exp. Psychol.* 31 843–856. 10.1037/0096-1523.31.5.843 16262482

[B43] Venuti P. Senese V. P. (2007). Un questionario di autovalutazione degli stili parentali: uno studio su un campione italiano. *G. Ital. Psicol.* 34 677–698. 10.1421/25224

[B44] Vosskühler A. Nordmeier V. Kuchinke L. Jacobs A. M. (2008). OGAMA (Open Gaze and Mouse Analyzer): open-source software designed to analyze eye and mouse movements in slideshow study designs. *Behav. Res. Methods* 40 1150–1162. 10.3758/BRM.40.4.1150 19001407

[B45] Zwaigenbaum L. Bryson S. Rogers T. Roberts W. Brian J. Szatmari P. (2005). Behavioral manifestations of autism in the first year of life. *Int. J. Dev. Neurosci.* 23 143–152. 10.1016/j.ijdevneu.2004.05.001 15749241

